# Dual Arm Co-Manipulation Architecture with Enhanced Human–Robot Communication for Large Part Manipulation

**DOI:** 10.3390/s20216151

**Published:** 2020-10-29

**Authors:** Aitor Ibarguren, Iveta Eimontaite, José Luis Outón, Sarah Fletcher

**Affiliations:** 1 Industry and Transport Division, TECNALIA, Basque Research and Technology Alliance (BRTA), 20009 San Sebastián, Spain; joseluis.outon@tecnalia.com; 2 School of Aerospace, Transport and Manufacturing, Cranfield University, Cranfield, Bedfordshire MK43 0AL, UK; Iveta.Eimontaite@cranfield.ac.uk (I.E.); s.fletcher@cranfield.ac.uk (S.F.)

**Keywords:** human–robot interaction, co-manipulation, human–robot interface, assistant robots

## Abstract

The emergence of collaborative robotics has had a great impact on the development of robotic solutions for cooperative tasks nowadays carried out by humans, especially in industrial environments where robots can act as assistants to operators. Even so, the coordinated manipulation of large parts between robots and humans gives rise to many technical challenges, ranging from the coordination of both robotic arms to the human–robot information exchange. This paper presents a novel architecture for the execution of trajectory driven collaborative tasks, combining impedance control and trajectory coordination in the control loop, as well as adding mechanisms to provide effective robot-to-human feedback for a successful and satisfactory task completion. The obtained results demonstrate the validity of the proposed architecture as well as its suitability for the implementation of collaborative robotic systems.

## 1. Introduction

The emergence of collaborative robotics changed the development of robotic solutions drastically for cooperative tasks. Industrial environments offer an interesting scenario for collaborative robotics, an environment where robots could act as assistants to operators [1,2], helping them in their usual tasks. Even so, the successful development of cooperative operations between the operators and robots gives rise to many challenges. Beginning from the low-level robot control [3] and ending with the social and acceptance aspects of these kinds of applications [4], many facets must be tackled during the implementation phase.

In cooperative manipulation tasks, one key aspect which is shared among almost all of them is the exchange of implicit and explicit information between both actors. For example, two operators are able to transport and place large parts with few or no visual information of their partner, using mainly the feedback of the forces sensed during the manipulation to adapt their trajectories and fulfill the task, adding extra information only when required. Even so, it is important choosing the most suitable cues for this information exchange to ensure successful completion of the task.

This paper presents a dual arm co-manipulation architecture for large part manipulation with enhanced human–robot communication capabilities. The proposed approach is based on three key elements: (1) Human driven co-manipulation, (2) coordination of dual arm robots and adaptation of trajectories to unexpected events, and (3) robot-to-human feedback for successful task completion.

The presented architecture tackles these three elements, defining a new scheme for dual arm co-manipulation tasks. This architecture pays special attention to the psychological aspects of the task, which is reflected in the inclusion of user studies in the architecture design and development phase. The implementation and testing of the architecture shows its suitability for cooperative industrial applications.

The paper is organized as follows. Section 2 provides information about related work. Section 3 presents the proposed architecture. Details about the low-level robot control and coordination are provided in Section 4. Section 5 gives information about data management for feedback generation. Section 6 presents the process carried out in the development of the *user interface*. Details about the implementation of the architecture are given in Section 7. Finally, Section 8 contains information about the conclusions and future work.

## 2. Related Work

Human–robot manipulation is a recurrent research topic, with multiple scenarios and approaches proposed. From classical scenarios with standard robotic manipulators [5], to the appearance of humanoid robots [6], many works about co-manipulation can be found in the literature.

Within the different topics posed in human–robot collaboration, force control is one of the most studied fields, with many approaches and algorithms to take advantage of the force based interaction. Lichiardopol et al. [3] propose a control scheme for human–robot co-manipulation with a single robot, where the system estimates an unknown and time-varying mass as well as the force applied by the operator. Dimeas and Aspragathos [5,7] pose a method to detect unstable behavior and stabilize the robot with an online adaptation of the admittance control gains, adding reinforcement learning to estimate parameters for effective cooperation. Peternel et al. [8] propose an approach for co-manipulation tasks such as sawing or bolt screwing through a human-in-the-loop framework which integrates online information about the human motor function and manipulability properties.

The use of Artificial Intelligence also helps improving co-manipulation applications, adding mechanisms to tune and optimize control models. Su et al. [9] propose the use of a recurrent neural network (RNN) to perform the trajectory control of redundant robot manipulators. Roveda et. al. [2,10] also propose the use of a neural network to optimize the control parameters, implementing a cooperative fuzzy-impedance control with embedded safety rules to assist human operators in heavy industrial applications while manipulating unknown weight parts. Moreover, Deep Learning algorithms have also been used for the identification of robot tool dynamics [11], allowing a fast computation and adding noise robustness.

Additionally, the use of predefined trajectories and guides for dual arm and co-manipulation tasks appear in different research works. Gan et al. [12] present a position/force coordination control for multi-robot systems where an object-oriented hierarchical trajectory planning is adopted as a first step of a welding task. Jlassi et al. [13] introduce a modified impedance control method for heavy load co-manipulation where an event controlled online trajectory generator is included to translate the human operator intentions into ideal trajectories. Following the topic of trajectory generation, Raiola et al. [14] propose a framework to design virtual guides through a demonstration using Gaussian mixture models.

Even so, few approaches include human and social factors in the system design. From the user point of view any new technology needs to be accepted by the workforce to be effective. Lack of trust can be caused by a lack of transparency in robot behaviour as shown in the work of Sanders et al. and Wortham and Theodorou [15,16]. People are likely to feel more comfortable and confident working with a robot if they know how it behaves and can anticipate what it will do next. In fact, Hayes and Scassellati [4] suggest that efficient communication between humans and robots is impossible without mutual understanding of each other behavior and appropriate expectations.

The robot’s action, communication, and transparency not only can increase task performance according to Lakhmani et al. [17], but operators’ well-being in terms of mental workload and situation awareness as noted by Hayes and Shah [18]. Furthermore, Sanders et al. [15] propose that a consistent and constant flow of information can have a positive impact on trust. Trust is essential for efficient task completion; according to Wright et al. [19], too little trust can result in technology rejection, while too much trust can lead to complacency. In situations with low levels of trust, mental workload and cognitive demand increase due to monitoring of the robot’s performance. Such increases result in less cognitive capacity left for monitoring the environment and complying with safety procedures as shown in Chen et al. and Saxby et al. [20,21]. In general, robot behavior transparency can have impacts on individual performance, trust, mental workload, and user experience. Studies establishing recommendations for human robot interaction and communication should assess these factors.

As an example of the previously exposed importance of the human factors, Weiss et al. [22] present a work where case studies are conducted during the use and programming of collaborative robots in industrial environments, adding an anthropocentric dimension to the work.

## 3. Proposed Architecture

As posed in the introduction, the aim of the presented work is to develop a dual arm robotic system able to assist human operators in manipulation tasks with large parts. The collaborative nature of this manipulation task raises many challenges, making it necessary to tackle different aspects ranging from robot control to human–robot interaction. In this sense, the presented work pays special attention to the human factors of the task in order to include the most suitable cues for an efficient and understandable information exchange between robot and human.

In the first step of the development, the large part transportation process has been analyzed in order to understand how humans perform it and extract the basic elements of the task, as well as the requirements to be transferred to the robot:

- During the transportation of large parts by humans, both actors agree (implicit or explicitly) on an approximate trajectory, which will be the basis for the transportation process. Following this premise, robots will manage a *nominal trajectory* which can be manually defined by users or generated automatically (e.g., using artificial vision).
- When humans transport large parts along a previously agreed path, any of them are able to deform this *nominal trajectory* in order to adapt the process to any unexpected event. In these cases, both actors are able to adapt their movements in a coordinated way without any prior knowledge, just based on the sensed forces. The implementation of *impedance control* [23,24] is proposed to mimic this behavior.
- The premise of this implementation is that robots act as assistants to the human. Taking this into account, robots will only advance in a trajectory when the operator moves the part along the defined path. Therefore, the operators will always play a master role in the co-manipulation task.
- As we are working with a dual arm robotic system, both arms need to move with a degree of coordination. Even so, this coordination will not be totally tight as in traditional robotics, as large part manipulation may require the adaptation of both robots due to uncertainties like the deformation of the objects or the human factor.
- During the part manipulation, besides the force feedback, humans exchange additional feedback as voice commands or gestures. It will be necessary to investigate how to include these cues in the robotic system.

To fulfill the previously presented requirements, a three-layer architecture is proposed:

- **Guidance Control Layer:** This initial layer is in charge of the low-level control of the robots, implementing a *Trajectory Driven Guidance with Impedance Control*.
- **Guidance Information Management Layer:** This second layer collects real time data of the *Guidance Control Layer* and generates meaningful information to be used as robot-to-human feedback.
- **User Interface Layer:** This last layer is the one in charge of presenting the guidance feedback to operators, using different cues to this end.

**Figure 1:**
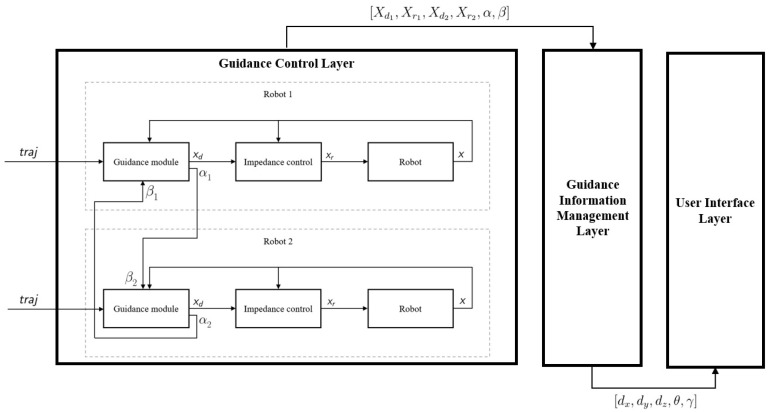
Architecture for dual arm co-manipulation.

This three-layer architecture allows the co-manipulation task to be performed, devoting specific modules to the control of the dual arm robot control and to the preparation and presentation of information for human–robot interaction. It covers all functionality from low-level control to high-level interaction feedback. Figure 1 illustrates the presented architecture.

The following sections provide further information about the different layers and their features.

## 4. Guidance Control Layer

The aim of this initial layer is to implement a control algorithm that allows the addition of a nominal trajectory to the impedance control while maintaining the coordination between two robot arms.

The main idea of the algorithm is to follow a provided trajectory as an operator guides the robot: the robot will transport the part smoothly along the trajectory while the robot will increase the resistance in the directions orthogonal to the nominal path. Additionally, impedance control is added to allow deformations on the path. It will provide some freedom to deform the robot path to the user as long as it guides the robot near the nominal trajectory. Besides the impedance control parameters, a set of trajectory points will also be used as input, points that will be linearly interpolated to generate the paths.

Specifically, the algorithm implements a two step control scheme for each robot. In a first step, the *Guidance module* calculates the next trajectory pose $X_{d}$ based on the nominal trajectory, current robot pose, and percentage of trajectory covered by both arms. In a second step, the *Impedance control module* modifies this pose in order to obtain a compliant behavior, calculating reference pose $X_{r}$.

The following sections provide information about the *Guidance module* and *Impedance control module*. Further details on this algorithm can be found in the work of Ibarguren et al. [25].

### 4.1. Guidance Module

The initial module of the control algorithm is based on a *Trajectory Driven Guidance with Impedance Control* and is in charge of calculating the set point sent to the *Impedance control module*. Based on a set of trajectory poses provided as input to the algorithm, it iterates along with the different segments of the trajectory. Specifically, these are the steps followed by the control algorithm to define the next set point (theoretical pose of each robot arm) in each loop based on an initial segment pose A and the end segment pose B:

- Project the current robot pose X in the vector $\vec{X_{d}^{i}B}$, where $X_{d}^{i}$ is the current setpoint and B is the end of the current segment of the trajectory.
  (1) $$\vec{P}=\frac{\vec{X_{d}^{i}X}\;\cdot \;\vec{X_{d}^{i}B}}{|\vec{X_{d}^{i}B}{|}^{2}}\;\vec{X_{d}^{i}B}$$
- At this step, the corrected advance vector $\vec{P_{c}}$ is calculated using the projection vector $\vec{P}$ and correction factor μ. It allows reducing the advance when the robot’s trajectory coverage is above the other robot’s, and increasing this advance otherwise.
  (2) $$\vec{P_{c}}=\mu \;\vec{P}$$
  The correction factor μ is calculated using values α, β, and λ, where α is the percentage of the trajectory covered by the robot arm, β is the percentage of the trajectory of the other robot arm, and parameter λ allows to tune this correction factor, adjusting the increase and decrease rate. If λ takes high values, the robot that has covered less trajectory percentage will be boosted (a maximum μ of 2) while the robot with greater trajectory percentage covered will be dampened (a minimum μ of 0). Otherwise, if λ is set to 0, there will not be any kind of coordination between the robots and the value of μ will always be 1.
  Additionally, the direction of the projection vector $\vec{P}$ is checked; if the vector points backwards the correction factor μ is set to 0 to avoid reverse movements.
  (3) $$\mu =\left\{\begin{array}{cc} 0, & \mathrm{if}\;\;\;\;\vec{P}\cdot \vec{X_{d}^{i}B}<0\\ 2-\frac{2}{1+e^{-\lambda (\alpha -\beta )}}, & \mathrm{otherwise} \end{array}\right.$$
- The new translation vector $\vec{X_{d}^{i+1}}$ is calculated as
  (4) $$\vec{X_{d}^{i+1}}=\vec{X_{d}^{i}}+\vec{P_{c}}$$
  while quaternion $Q^{i+1}$ is interpolated between the rotations of poses A and B using the *spherical linear interpolation* [26] as
  (5) $$Q^{i+1}=SLERP\left(Q_{A},Q_{B},w\right)$$
  (6) $$SLERP\left(Q_{A},Q_{B},w\right)=Q_{A}\;exp\left(w\;log\left(Q_{A}^{-1}\;Q_{B}\right)\right)$$
  where *w* is calculated as
  (7) $$w=\frac{|\vec{{\mathrm{AX}}_{d}^{i+1}}|}{|\vec{\mathrm{AB}}|}$$
- Finally, the desired set point $X_{d}$ is composed using translation vector $\vec{X_{d}^{i+1}}$ and rotation matrix $R^{i+1}$ created from quaternion $Q^{i+1}$ as
  (8) $$X_{d}=\left[\begin{array}{c} \begin{array}{cc} R^{i+1} & \vec{X_{d}^{i+1}}\\ 0 & 1 \end{array} \end{array}\right]$$

This new pose $X_{d}$ is sent to the *Impedance control module* to be used as the set point.

### 4.2. Impedance Control Module

In the second step, a compliant Cartesian behavior is added to each robotic arm implementing Cartesian impedance control [27,28]. The *Impedance control module* allows to establish a mass–damper–spring relationship between the Cartesian position Δx and the Cartesian force F, the following formula is applied,

(9) $$F=M\Delta \ddot{x}+D\Delta \dot{x}+K\Delta x,$$

where M, D, and K represent the virtual inertia, damping, and stiffness of the system, respectively.

To calculate the reference pose $X_{r}$, based on the previously calculated set point $X_{d}$ and the sensed force vector F,

(10) $$X_{r}=X_{d}-\frac{\Delta F}{M\Delta t^{2}+D\Delta t+K}\;,$$

where ΔF represents the difference between the desired contact force and the actual one.

This pose $X_{r}$ is sent to the robot for the execution of the dual arm co-manipulation trajectory.

## 5. Guidance Information Management Layer

As stated previously, this second layer collects real time information from the *Guidance Control Layer* and manages it in order to generate meaningful information to be used as robot-to-human feedback. Therefore, this step converts raw information provided by the control layer into human-understandable data.

The *Guidance Information Management Layer* receives input vector G containing the following values,

(11) $$G=[X_{d_{1}},X_{r_{1}},X_{d_{2}},X_{r_{2}},\alpha ,\beta ],$$

where $X_{d_{1}}$ and $X_{d_{2}}$ represent the set point of each robot, $X_{r_{1}}$ and $X_{r_{2}}$ define the reference pose of both robots after applying the Cartesian impedance control, and α and β are the trajectory percentage covered by the robots.

Based on this information, this layer calculates metrics about the deviation in the trajectory, quality of the guidance and the overall trajectory percentage covered. Specifically, the trajectory deviation $D=[d_{x},d_{y},d_{z}]$ is calculated as

(12) $$D=\left[d_{x},d_{y},d_{z}\right]=\frac{\left(\vec{X_{d_{1}}}-\vec{X_{r_{1}}}\right)+\left(\vec{X_{d_{2}}}-\vec{X_{r_{2}}}\right)}{2},$$

where $\vec{X_{d_{1}}}$ and $\vec{X_{d_{2}}}$ represent the translation part of both setpoints and $\vec{X_{r_{1}}}$ and $\vec{X_{r_{2}}}$ are the translation part of the reference poses of both robots.

To quantify the quality of the guidance θ, the following equation has been defined,

(13) $$\theta =\lambda_{traj}\;min\left(1,\frac{\left|\alpha -\beta \right|}{m_{traj}}\right)+\lambda_{dev1}\;min\left(1,\frac{∥\vec{X_{d_{1}}}-\vec{X_{r_{1}}}∥}{m_{dev}}\right)+\lambda_{dev2}\;min\left(1,\frac{∥\vec{X_{d_{2}}}-\vec{X_{r_{2}}}∥}{m_{dev}}\right),$$

(14) $$\lambda_{traj}+\lambda_{dev1}+\lambda_{dev2}=1$$

where values $m_{traj}$ and $m_{dev}$ are the maximum trajectory percentage difference and trajectory deviation allowed, respectively, acting as a threshold. Values $\lambda_{traj}$, $\lambda_{dev1}$, and $\lambda_{dev2}$ are weighting factors that allow defining which of the measures (trajectory percentage difference or trajectory deviation of each robot) have more impact in the quality of the guidance. Therefore, the equation will provide a value ranging from 0 to 1, where 0 indicates a perfect trajectory guidance and 1 indicates a guidance error over the limits set through the different parameters.

Finally, the mean of the covered trajectory percentage γ is calculated as

(15) $$\gamma =\frac{\alpha +\beta }{2}.$$

These previous equations will calculate vector U as

(16) $$U=[d_{x},d_{y},d_{z},\theta ,\gamma ],$$

where values $d_{x}$, $d_{y}$, and $d_{z}$ contain information about the guidance deviation; θ defines the quality of the guidance process; and γ is the mean trajectory percentage covered during the process.

This vector U is the data that will be used as input in the *User Interface Layer* to generate the appropriate cues for the robot-to-human interaction and feedback.

## 6. User Interface Layer

Information communication is one of the essential factors for developing successful human–robot interaction. Therefore, this *User Interface Layer* aims to provide an interface able to generate suitable and understandable cues to communicate the status of the co-manipulation task. Although some research suggests that modality of information communication (audio, text, and graphic) does not affect trust and user experience as shown in [15], Selkowitz et al. [29] have found that the use of a graphic information display does not significantly increase user workload. In addition to possible positive effects on workload, the graphic modality has the benefit of requiring little experience and training to use [30], and it can be beneficial for people with different information processing abilities due to, for example, dyslexia as discussed in [31]. Another advantage is that, in most cases, universally understood symbols can be used to provide support to people from different countries and cultures as considered by Ben et al. [32].

To identify and select the most effective and understandable cues, two user studies were carried out. These studies will help in the development and design of the *user interface* as they will identify the most effective way of presenting the information generated in the *Guidance Information Management Layer*. The psychological and performance impact of the developed human–robot collaboration communication was investigated over these two studies.

In the initial phase of the user interface design process, a number of possible cues were identified. Individuals in a team communicate by gaze and non-verbal communication [33,34] and to replicate this, the current study used an avatar representing the robot and added head movements to indicate trajectory deviation. In addition, trajectory deviation and trajectory percentage parameters were introduced as graphical symbols dynamically providing information about the task to the user. Finally, the user interface display used a background with a universally known paradigm of a traffic light to establish how far from the optimal path the robot is (from green color indicating on an optimal path to red indicating a strong deviation).

The following sections provide further details about the two user studies.

### 6.1. User Study 1

**Figure 2:**
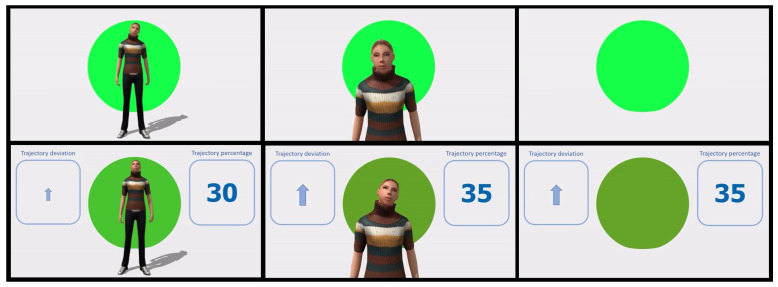
Six tested user interfaces.

The main aim of *User Study 1* was to investigate which user interface is the most effective in indicating the robot behavior and how these user interfaces can be improved. To achieve this aim, six versions of the user interface were used in an online study with qualitative and quantitative questions. Six experimental conditions in the form of 20 s video clips of an operator and a robot collaborating were presented to all participants in a counterbalanced order. The six different visual user interfaces indicating what the robot is communicating to the operator are presented in Figure 2 from top left to bottom right as follows.

- Full body avatar and background color (A)
- Avatar torso and background colour (B)
- Background colour (C)
- Dashboard, full body avatar and background color (D)
- Dashboard, avatar torso and background colour (E)
- Dashboard and background colour (F)

The study collected quantitative and qualitative data. The quantitative question asked participants to rate user interface clarity on a scale from 0 “Extremely unclear” to 100 “Extremely clear”. This question was split into several sub-parts on each of the core elements of the user interface depending on the condition (avatar face, avatar posture, background, trajectory deviation, and percentage deviation). The mean of the answers for each core element was calculated to produce an overall clarity rating. The data had a normal distribution and was further analyzed with a repeated measures ANOVA. The qualitative questions asked the participants to describe what they thought the user interface was trying to communicate to the operator and how it could be clarified. Twenty-eight participants provided qualitative responses for open-ended questions; however, only 18 of them answered all the quantitative questions allowing further inferential analysis. Eleven participants indicated their gender as male, five as female, three reported as “other”, and nine did not answer the question. The average age of participants was 33.11 years (SD = 9.49) with ten participants not providing their age. Three participants reported they came from the manufacturing industry, twelve from an academic environment, one from “other—construction”, while ten did not respond where they were working. The study was approved by the Cranfield University Research Ethics Committee.

The user interface information clarity was significantly different between conditions (F(2.37,85)=8.80,p≤0.001). Post hoc comparison with Bonferroni correction between conditions indicated that the clarity was significantly higher for the Dashboard and background color (F) user interface compared to all user interfaces with an avatar, but there was no significant difference with the Background (C) user interface (Table 1). On the other hand, the Avatar torso and background color (B) user interface was rated significantly lower on clarity compared to all other conditions except the Full body avatar and background color (A) and the Dashboard, full body avatar, and background color (E) user interfaces (see Table 1 for the significance levels of all comparisons).

Looking at the participants’ preferences, 75% of participants preferred the user interface with only the Dashboard and background (F), 15% preferred the Dashboard, avatar torso and background color (E), 5% preferred only the Background (C) and 5% preferred the Avatar torso and background color (B). The full body size avatar (with or without dashboard (A and D)) was not chosen by any participant as a preferred user interface.

To shed some light on participant ratings on user interface clarity, participants’ answers to the open-ended questions were analyzed further:

- Participants’ opinions about the use of the avatar were split: Some participants appreciated that the avatar “makes (it) more comfortable to interact with a robot”, other participants expressed dissatisfaction with it: “I already have my partner that bosses me around at home, I don’t need another one in the workshop”. Participants communicated that having only the avatar torso was more useful than the full body size avatar as the legs do not convey any task related information. Furthermore, the avatar’s head movements were subtle and not all participants understood/noticed them; therefore, for future development more pronounced head movements were suggested.
- In relation to the other user interface features, participants indicated that the background circle should be accompanied by a benchmark scale indicating trajectory from “good” to “bad”.
- Participants also commented that it might be useful to introduce commands or a feedback display to keep the communication less ambiguous. Some of the participants indicated that this could be done via audio feedback to the user.

These suggestions were included in the user interface in *User Study 2*.

### 6.2. User Study 2: Laboratory Results

**Figure 3:**
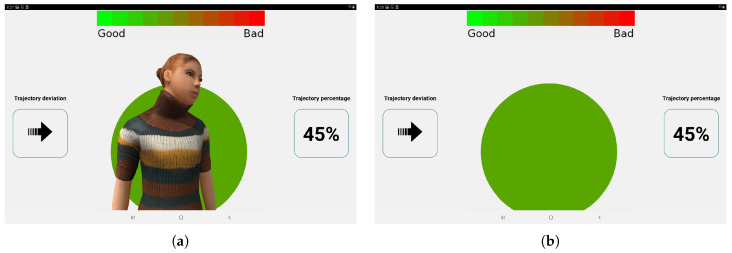
User interfaces for *User Study 2*. (**a**) The dashboard with the avatar (torso) (**b**) The dashboard without the avatar.

Two types of the user interface were selected from *User Study 1* and adjusted according to the participant suggestions; specifically, the dashboard with the avatar (torso) (Figure 3a) and the dashboard without the avatar (Figure 3b). The two user interfaces included the following cues:

- Dashboard with the deviation from the trajectory and trajectory percentage.
- Avatar with head movements to indicate the deviation from the trajectory.
- Background color indicating the deviation from the trajectory.
- Voice commands indicating deviations from the trajectory.

**Figure 4:**
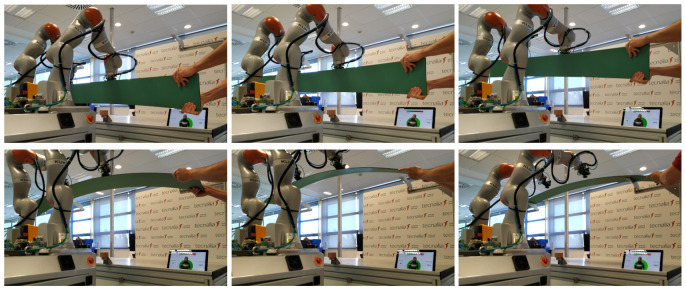
Transporting a carbon fiber part from fixtures to shelf during *User Study 2* experiments.

The main aim of *User Study 2* was to determine how the selected user interfaces affect collaborative task performance and participant well-being. The task required the participant and the robot to collaboratively remove a component from a shelf and place it in a fixture on the desk and then move back to the shelf (Figure 4). Thirteen participants took part in the study of which ten were males, two were females and one did not indicate their gender. The average age was 36.85 years (SD = 7.65). Seven participants indicated that they work with robots every day, two on a regular basis, three responded that they work with robots sometimes but not on a regular basis, and one participant said that they have never worked with robots. All participants took part in all conditions: two experimental conditions and the control condition with no user interface. The conditions were counterbalanced and the study assessed behavioral parameters (deviation from the optimal path and time to complete the tasks), and collected self-report data (Trust in Industrial Robot [35], NASA TLX [36], and User Experience Questionnaire [37]). This paper will focus on the behavioral and the User Experience Questionnaire results as the NASA TLX and Trust in Industrial Robot scales did not indicate any significant differences (p>0.05). Additional qualitative questions relating to the user interface focused on what participants found the most useful for task completion and their suggestions for improvement of user interface clarity. The study was approved by the Cranfield University Research Ethics Committee.

To investigate how different user interfaces affect human performance efficiency, the mean task completion time and the mean deviation from the optimal trajectory were compared between conditions with a nonparametric Friedman’s ANOVA as the data was not normally distributed. The mean task completion time was measured in seconds. The analysis showed a trend difference in the mean deviation $\left(X^{2}\left(2\right)=4.77,p=0.092\right)$, but no significant difference in the completion time $\left(X^{2}\left(2\right)=0.15,p=0.926\right)$, see Table 2. Further investigation was performed on the mean deviation from the optimal trajectory which showed a lower deviation in the avatar condition compared to the control condition at a trend significance level (Z=1.71,p=0.090), but the differences between the no-avatar condition and the control condition or the avatar and the no-avatar condition were not significant (Z=1.36,p=0.185) and (Z=0.14,p=0.906).

**Figure 5:**
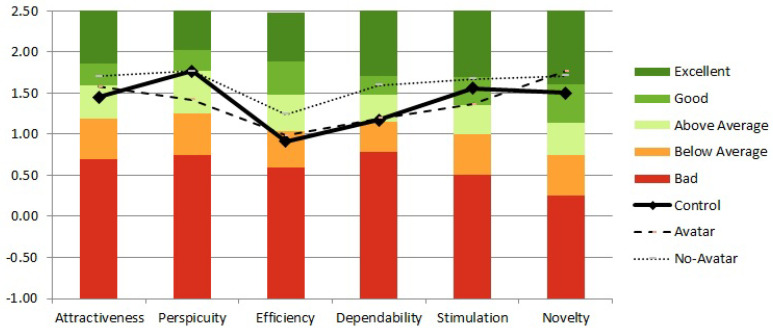
Reported user experience across six UEQ factors as a function of the experimental condition.

Finally, the subjective evaluation of participants’ experience was measured with the User Experience Questionnaire (UEQ). Although the results were not significant $\left(X^{2}\left(5,20.84\right)\;\leq \;1.62,p\;\geq \;0.200\right)$, the score means across the factors between the conditions indicate that participants evaluated the user interface without the avatar relatively higher than the other interfaces, while the user interface with the avatar scored highest on the novelty factor. It is important to indicate that the control condition and the user interface with the avatar both ranked below average on the efficiency factor (Figure 5).

In their qualitative feedback, 38% of the participants indicated that audio information was the most useful and another 38% of the participants indicated the trajectory deviation and trajectory percentage were the most useful. Fifteen percent of the participants indicated that they would include depth information for the trajectory deviation, one participant asked for more detailed audio information to guide the movement, while another participant explained that they used mainly audio information to complete the task. Fifteen percent of the participants indicated that they did not use the avatar during the task as, according to them, it did not provide useful information. This qualitative information confirms the User Experience Questionnaire results indicating that participants found the user interface with trajectory deviation and trajectory percentage the most useful. However, behavioral information from the task suggest that the presence of the avatar can reduce the deviation from the optimal trajectory.

Based on the obtained results, it was decided to maintain both user interfaces in the presented robotic system, allowing the operators to choose between both options based on their preferences.

## 7. Implementation

**Figure 6:**
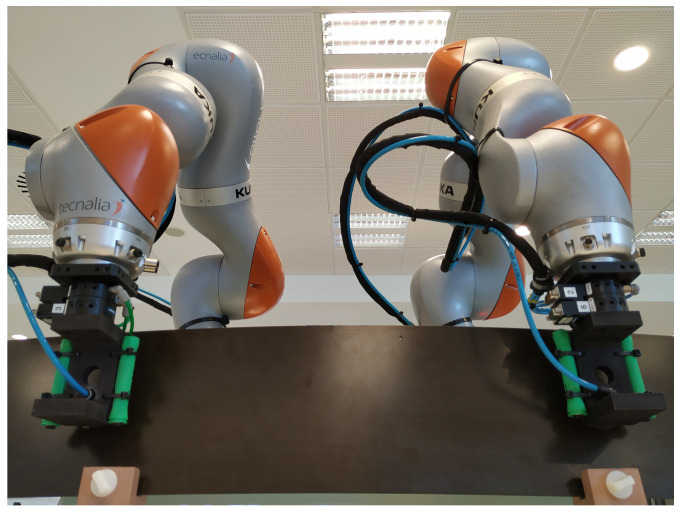
Set-up with two Kuka LBR iiwa robots.

The proposed robotic system has been implemented using a setup of two *Kuka LBR iiwa* robots with a payload of 7 kg for each arm, mounted on a mobile platform. The mobile manipulator includes an additional PC connected to both robot controllers. The robots are equipped with automatic tool exchangers and vacuum cups to allow grasping different types of large objects and parts. An additional IO module is also available to manage the tool exchangers as well as the suction of the vacuum cups. Figure 6 shows the set-up of the robots.

A tablet has been used as interface for the guidance system. The selection of a tablet allows the mobility required for this kind of applications, as operators can transport it and place it in the most suitable placement for each co-manipulation task.

From the software point of view, all the computation of the *Guidance Control Layer* has been implemented in Java in the robot controllers. The *Impedance control module* makes use of Kuka Sunrise’s Smart Servo library to close the control loop. Additionally, the external PC acts as bridge between both robots by means of several custom ROS nodes written in C++ which manage the connections and exchanges the trajectory information. Besides, this same PC runs the *Guidance Information Management Layer* as well as the *User Interface Layer*, which acts as HTML5 server providing the web interface to the tablet.

Finally, the tablet only acts as user interface, displaying the different cues based on the values sent from the external PC using any web browser installed on it. The implementation allows the use of multiple devices at the same time, therefore it could be possible to include several operators interacting with the robot at the same time.

## 8. Conclusions and Future Work

This paper presents a novel architecture for dual arm co-manipulation with an enhanced human–robot communication capabilities. The architecture addresses different aspects of a co-manipulation task, from the control algorithm to the user interface, paying special attention to the user experience and psychological aspects of the human–robot collaboration.

Initially, a control algorithm for the dual arm robots is presented, implementing a *Trajectory Driven Guidance with Impedance Control*. This algorithm allows guiding the robot along some virtual guides during the part transportation phase. The inclusion of an impedance control module adds flexibility as operators are able to deform the theoretical trajectory in order to face unexpected events or to correct errors.

Additionally, the architecture provides different modules to manage the information exchange between the robot and the human. The aim of these modules is to provide effective and understandable feedback for the completion of the part transport task. To improve the design of the user interface and select the most suitable cues, two *User Studies* have been carried out. The findings of both studies provide further insight on how a robot could communicate the task related information to the human. The user feedback on the clarity of the user interface, their needs and requirements to make the collaboration more transparent (Study 1) allowed to adjust the user interface and conduct a behavioral experiment (Study 2). The behavioral results of the actual human–robot interaction task show that the presence of the user interface has an impact on the performance, improving the precision of the guidance using the most suitable cues.

Even so, several aspects of the architecture can be further developed and improved as a future work. On the one hand, the impedance control parameters could be modified in execution time based on the human behavior in order to offer a more human-like manipulation experience. Furthermore, it would be interesting to add mechanisms to detect collisions (with external elements or between the robot arms) to ensure a safe human–robot interaction. On the other hand, suggestions made by participants in *User Study 1* and *2* could be implemented. These suggestions include presenting movement depth information or including the visual representation of the component, as well as recommendations to further develop audio information. Additionally, the inclusion of voice commands from operator to robot (e.g., to start/stop the guidance) would be an interesting addition to the co-manipulation tasks. The voice commands would be especially important during the manipulation of the large parts where it is difficult to use physical devices to input commands as parts need to be carried by both hands. Therefore, the inclusion of speech recognition and conversational agents would help to create a seamless human-to-robot interaction channel.

## Figures and Tables

**Table 1:** Post hoc statistics and descriptive information between all experimental conditions.

	Avatar Torso and Background	Dashboard, Avatar Torso and Background	Dashboard and Background	Background	Full Body Avatar and Background	Dashboard, Full Body Avatar and Background
Avatar torso and background		*p* = 0.043	*p* = 0.007	*p* = 0.025	p≥0.999	*p* = 0.072
Dashboard, avatar torso and background			*p* = 0.021	p≥0.999	*p* = 0.463	p≥0.999
Dashboard and background				p≥0.999	*p* = 0.018	*p* = 0.034
Background					*p* = 0.017	p≥0.999
Full body avatar and background						*p* = 0.378
Mean (SD)	20.63 (4.11)	31.42 (4.95)	45.85 (6.82)	42.39 (7.63)	21.87 (4.28)	31.27 (4.49)

**Table 2:** Means (SD) across three experimental conditions on the behavioral and user experience questionnaire measures.

	Control		Avatar		No-Avatar	
	Mean	SD	Mean	SD	Mean	SD
**Behavioral data**						
Completion time (sec)	42.71	10.74	45.57	12.56	43.85	11.68
Mean deviation	46.58	12.08	39.69	7.30	42.15	13.18
**User Experience Questionnaire**						
Attractiveness	1.45	0.69	1.58	0.98	1.70	0.78
Perspicuity	1.77	0.85	1.42	0.97	1.77	0.75
Efficiency	0.92	0.71	0.98	0.88	1.24	0.86
Dependability	1.17	0.70	1.19	0.74	1.60	0.67
Stimulation	1.56	0.46	1.3	1.10	1.67	0.74
Novelty	1.50	0.87	1.77	0.89	1.71	0.97
